# Extending the theory of planned behavior in mathematics education: teachers’ innovative pedagogical knowledge, classroom climate, and gender differences in students’ innovative attitudes

**DOI:** 10.3389/fpsyg.2026.1799251

**Published:** 2026-06-15

**Authors:** Khaled Ben-Motreb

**Affiliations:** Department of Curriculum and Instruction, College of Education, King Faisal University, Al-Ahsa, Saudi Arabia

**Keywords:** classroom climate, creativity and innovation, gender differences, innovative teaching, mathematics education, Saudi Arabia

## Abstract

Fostering students’ innovative capacities in mathematics is increasingly recognized as both a psychological and educational priority, yet little is known about how mathematics teachers’ innovative pedagogical knowledge and the classroom climate are jointly associated with students’ innovative attitudes, particularly in gender-segregated systems. Guided by the Theory of Planned Behavior (TPB), this study surveyed 300 first-year secondary mathematics students in Saudi Arabia (150 males, 150 females) using validated instruments that assessed perceptions of teachers’ innovative pedagogical knowledge, classroom climate, and students’ innovative attitudes toward learning mathematics. Using a TPB-informed framework, the study examined how instructional and classroom factors were associated with students’ innovative attitudes in mathematics learning. Analyses included correlations, independent-samples *t*-tests, mediation models, multiple regression, and hierarchical regression. Results indicated that teachers’ innovative pedagogical knowledge was significantly associated with students’ innovative attitudes in mathematics, with stronger effects among female students. For girls, classroom climate showed a modest indirect association between teachers’ innovative pedagogical knowledge and innovative attitudes, while no evidence of a comparable indirect pathway was observed for boys. Gender also moderated the strength of predictors, as both teacher knowledge and classroom climate showed stronger associations with female students. Collectively, these factors explained approximately 25% of the variance in female students’ innovative attitudes toward mathematics learning, compared with a smaller share for males. The findings highlight gender-related differences in how instructional practices and classroom environments are associated with students’ innovative attitudes in mathematics and emphasize the importance of professional development and gender-responsive classroom practices that support creativity and innovation in mathematics learning.

## 1 Introduction

Educational systems worldwide are under increasing pressure to prepare students who can think flexibly and solve complex problems in rapidly changing social, technological, and economic environments. International policy agendas consistently emphasize innovation not only as a cornerstone of 21st-century competencies but also as a psychological capacity that enables learners to adapt, remain motivated, and build resilience in knowledge-based economies. Within this global agenda, mathematics provides a unique platform for cultivating innovation: it demands logical reasoning while also requiring students to generate original, non-traditional solutions to both abstract and real-world problems. In this sense, mathematics education represents both a cognitive and a socio-emotional space where innovation can be nurtured.

While closely related, the constructs of creativity, innovation, and innovative attitudes are conceptually distinct in educational research. Creativity typically refers to the cognitive capacity to generate novel or original ideas, particularly in problem-solving contexts. Innovation, in contrast, involves the practical application or implementation of such ideas in real learning situations, such as adopting new strategies or alternative solution pathways. Innovative attitudes represent a different dimension: they reflect students’ dispositional openness and willingness to engage with non-traditional approaches to learning and problem solving. In mathematics education, innovative attitudes therefore capture students’ readiness to explore alternative reasoning strategies and to value creative approaches, even when they may not yet produce fully original mathematical solutions.

Teachers play a central role in shaping these outcomes. Their innovative pedagogical knowledge equips them to design classroom practices that stimulate creativity, foster supportive socio-emotional climates, and shape students’ innovative attitudes. Classic scholarship has long stressed the link between innovative teaching and creativity in classroom settings (Torrance, 1964; Amabile, 2012). More recent evidence similarly suggests that mathematics teachers who encourage fluency, flexibility, and originality are more likely to create classrooms that support innovative thinking and non-traditional problem solving.

Recent research in mathematics education increasingly highlights the role of the emotional climate in supporting mathematics learning (Rodríguez et al., 2020), while innovative classroom environments are associated with stronger problem-solving, with stronger effects among female students (Lu et al., 2025). Mathematical creativity has also been linked to open-ended tasks, modeling activities, and technology-supported learning environments that encourage students to explore alternative solution pathways (El Turkey et al., 2024; Ye et al., 2024; Lu et al., 2025). Similarly, lateral-thinking activities appear to support flexible mathematical reasoning (Shodig et al., 2025). At the same time, even teachers with strong conceptual knowledge of mathematical creativity may struggle to fully translate it into classroom practice, underscoring the importance of supportive environments in encouraging innovative pedagogy (Turan et al., 2025). Overall, previous research suggests that innovative teaching practices and supportive classroom climates jointly contribute to students’ engagement and innovation-related learning outcomes (Nkundabakura et al., 2023).

These international findings also resonate with evidence from diverse cultural contexts. Technology integration has been found to enhance students’ innovative capacities, with outcomes shaped by gender and culture (Alshahrani and Ally, 2016). Digital tools have been observed to foster creativity among youth (Singh and Alwaqaa, 2023). Analyses of TIMSS 2019 data further indicate that gender differences in teaching practices and classroom climate are reflected in students’ attitudes toward mathematics and science, with female students perceiving more supportive environments and demonstrating stronger innovation-related dispositions (Alnahdi and Schwab, 2023). Such results highlight that both cognitive and socio-emotional factors interact in shaping students’ attitudes and dispositions toward innovation.

Despite these contributions, important gaps remain. Much prior research has examined either teachers’ practices or classroom climate in isolation, often focusing on broad constructs such as general creativity or technology use rather than students’ innovative attitudes in mathematics specifically. Only a limited number of studies have explored how teachers’ innovative pedagogical knowledge and classroom climate work together, and even fewer have investigated gender differences in these relationships. The combined testing of mediation (through classroom climate) and moderation (through gender) is particularly rare, especially in gender-segregated secondary classrooms.

To address these gaps, the present study investigates: (a) the relationships between students’ perceptions of teachers’ innovative pedagogical knowledge, classroom climate, and their innovative attitudes; (b) gender-based differences in these associations; and (c) whether classroom climate mediates the influence of teacher knowledge, with gender moderating the overall framework. The study contributes by: (1) providing evidence from gender-segregated secondary mathematics classrooms; (2) testing mediation and moderation mechanisms together within a single design; and (3) offering practical insights for teacher development and classroom design. Theoretically, this research extends the Theory of Planned Behavior (TPB) into mathematics education by linking normative influences (teachers’ innovative knowledge) and perceived behavioral control (classroom climate) to students’ attitudes (Ajzen, 1991), with gender as a contextual moderator. Practically, it suggests strategies for professional development and classroom environments that are responsive to gender and capable of fostering innovation. Together, these contributions advance global understanding of how mathematics classrooms, viewed through both educational and psychological lenses, can prepare students for the challenges of the future.

## 2 Theoretical framework

This study is anchored in the Theory of Planned Behavior (TPB) (Ajzen, 1991), a psychological model that explains how attitudes, subjective norms, and perceived behavioral control interact to shape human behavior. Within educational psychology, TPB has been widely applied to understand students’ learning dispositions, motivational orientations, and behavioral intentions in classroom contexts. It offers a robust framework for examining how teacher-related and environment-related factors jointly influence students’ innovative attitudes in mathematics education. The present study adopts a TPB-informed framework representing a contextualized application of the Theory of Planned Behavior rather than a full structural test of the model. Ajzen’s (1991) model includes attitudes, subjective norms, perceived behavioral control, intentions, and behavior, the current investigation focuses on attitudes as the primary outcome of interest in mathematics learning contexts. This approach allows the study to examine instructional and classroom factors associated with students’ innovative attitudes while remaining theoretically grounded in the core logic of TPB. In this framework, the study conceptualizes the constructs as follows:

- Teachers’ innovative pedagogical knowledge is conceptualized as a form of subjective norm, signaling to students that innovation is expected, valued, and supported in mathematics learning. Conceptually, this construct refers to teachers’ instructional capacity to employ creative and non-routine pedagogical strategies in mathematics classrooms. Such practices may encourage students to engage more openly in creative mathematical thinking.
- Innovative classroom climate can be interpreted as reflecting perceived behavioral control, capturing the extent to which the socio-emotional environment of the classroom enables or constrains students’ opportunities to act innovatively. This construct represents students’ shared perception of a supportive classroom environment that encourages experimentation and multiple solution strategies.
- Students’ innovative attitudes serve as the attitudinal outcome, representing their openness and readiness to adopt non-traditional, creative approaches to problem-solving in mathematics. Unlike the previous constructs, which describe instructional and environmental conditions, this construct reflects students’ individual dispositions toward innovative mathematical thinking.
- Gender is included as a contextual moderator, recognizing that the dynamics of gender-segregated schooling not only structure learning opportunities but also shape psychological processes related to motivation, self-efficacy, and responsiveness to classroom environments, as prior research in mathematics education has documented systematic gender differences in students’ responses to instructional practices and classroom socio-emotional environments.

By aligning these constructs with TPB, the study positions students’ innovative attitudes as the central outcome and investigates how teachers’ innovative knowledge and classroom climate function as psychological and contextual factors associated with students’ innovative attitudes. At the same time, gender is treated as a socio-cultural factor that moderates the strength of these pathways, thereby enriching the application of TPB in mathematics education through both educational and psychological lenses.

### 2.1 Conceptual model and hypotheses

Drawing on the Theory of Planned Behavior (TPB), this study formulates four hypotheses that integrate psychological and educational constructs within the context of mathematics classrooms. First, teachers’ innovative pedagogical knowledge is expected to be positively associated with students’ innovative attitudes, since exposure to innovative practices communicates valued social norms and shapes students’ motivational orientations and willingness to engage in creative learning (H1). Second, classroom climate is hypothesized to mediate this relationship, as supportive and engaging environments may help explain how teacher innovation is associated with students’ attitudes toward innovation (H2). Third, gender is expected to moderate these associations: given gender-segregated schooling and prior evidence of differential socio-emotional sensitivity to classroom environments, stronger effects are anticipated among female students (H3). Finally, teachers’ innovative knowledge and classroom climate are expected to jointly predict students’ innovative attitudes, with the magnitude of prediction varying by gender (H4).

**FIGURE 1:**
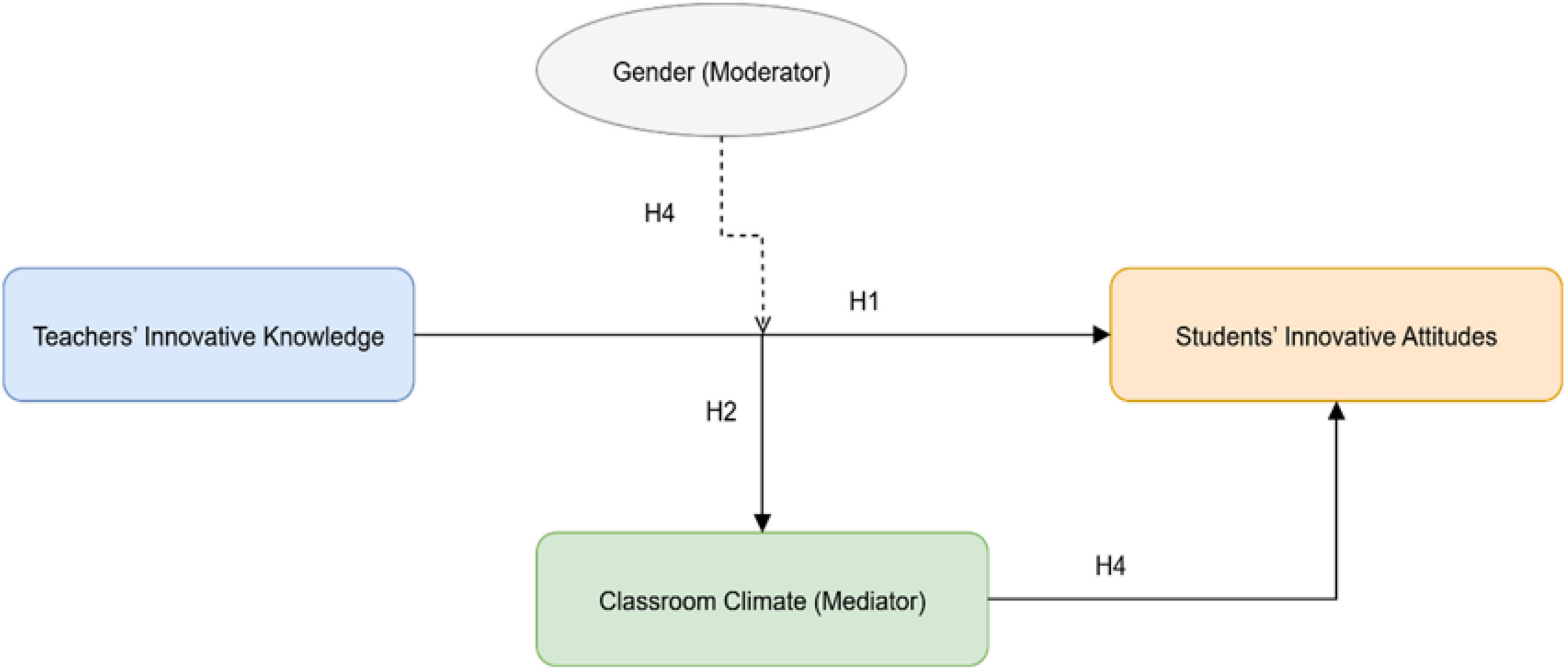
Conceptual model and hypotheses of the study based on the Theory of Planned Behavior (TPB).

As shown in Figure 1, the conceptual model specifies four hypotheses: (a) a direct association between teachers’ innovative knowledge and students’ innovative attitudes (H1); (b) a mediating role of classroom climate in this relationship (H2); (c) a moderating effect of gender on these associations (H3); and (d) a joint predictive effect of teachers’ innovative knowledge and classroom climate on students’ innovative attitudes (H4). These hypotheses are designed to provide both theoretical and practical insights into relationships and contextual factors and educational conditions that shape innovation in mathematics learning.

Teachers’ innovative pedagogical knowledge is hypothesized to directly predict students’ innovative attitudes (H1), with classroom climate mediating this relationship (H2). Gender moderates both associations (H3), while innovative knowledge and classroom climate are expected to jointly predict students’ innovative attitudes (H4).

Within this TPB-informed framework, teachers’ innovative knowledge and classroom climate are treated as instructional and contextual factors associated with students’ innovative attitudes. Correlation and regression analyses assess the direct associations (H1, H4), mediation analysis tests the indirect role of classroom climate (H2), and moderation analysis evaluates the moderating effect of gender (H3). This alignment links the theoretical framework with the empirical analyses used in the study.

## 3 Materials and methods

### 3.1 Research design

This study employed a quantitative descriptive–correlational design to investigate psychological and educational relationships among students’ perceptions of their mathematics teachers’ innovative pedagogical knowledge, the innovative classroom climate, and their innovative attitudes. The design further enabled the testing of two mechanisms central to psychological modeling: the mediating role of classroom climate and the moderating role of gender. A correlational approach was deemed appropriate as it allows the analysis of naturally occurring psychological variables (attitudes, perceptions, socio-emotional climate) in authentic school settings without experimental manipulation, thereby ensuring ecological validity.

### 3.2 Participants

The population included all first-grade secondary students in public schools within a medium-sized educational district in western Saudi Arabia during the 2024 academic year (approximately 12,000 students). The schools served students from diverse socioeconomic backgrounds typical of public education in the region. A stratified random sampling method was applied to ensure equal representation of male and female students, reflecting the structure of gender-segregated schooling in Saudi Arabia.

The final sample comprised 300 students (150 males and 150 females) from 10 schools (five for boys and five for girls). Krejcie and Morgan’s table confirmed that this sample exceeded the recommended size (*n* = 291) for populations over 10,000 at the 95% confidence level and a 5% margin of error, ensuring both representativeness and statistical adequacy.

### 3.3 Instruments

#### 3.3.1 Teacher innovative behavior scale

This instrument was designed to capture students’ perceptions of two key psychological–educational constructs: their mathematics teachers’ innovative pedagogical knowledge and the extent to which the classroom climate supported innovation.

It consisted of 20 items across two subscales. The first, Innovative Knowledge (10 items), assessed teachers’ ability to present mathematical concepts creatively and non-traditionally, for example through real-life applications or visual representations. The second, *Innovative Classroom Climate* (10 items), measured the socio-emotional climate of the classroom—whether it encouraged experimentation, open discussion, and multiple solution pathways.

Responses were collected using a five-point Likert scale (1 = Strongly Disagree to 5 = Strongly Agree). Items were adapted from prior validated instruments (Amabile, 2012; Lu et al., 2025), reviewed by five subject experts, and piloted with 30 students. Minor revisions ensured cultural alignment and clarity. Internal consistency was strong, with Cronbach’s alpha = 0.88 for the innovative knowledge subscale and 0.86 for the classroom climate subscale, reflecting robust psychometric reliability.

#### 3.3.2 Students’ innovative attitudes scale

This scale measured students’ attitude toward adopting creative and non-traditional approaches in mathematics learning.

It included 15 items assessing students’ preference for alternative problem-solving methods. Responses were recorded on a five-point Likert scale (1 = Strongly Disagree to 5 = Strongly Agree). Items were grounded in established theoretical models (Singh and Alwaqaa, 2023; Turan et al., 2025), reviewed by experts, and piloted with 30 students outside the main sample. Cronbach’s alpha reached 0.90, indicating excellent psychometric reliability.

### 3.4 Procedures and ethical considerations

Ethical approval was obtained from the Research Ethics Committee at King Faisal University (KFU-2025-ETHICS3607). Written informed consent was secured from students’ guardians, with assurances of confidentiality and the exclusive use of data for academic purposes. The study complied with international standards for psychological and educational research ethics, including the principles of the Declaration of Helsinki.

Data collection took place between October 1–15, 2024, during regular classroom sessions across ten public secondary schools (five boys’ schools and five girls’ schools) within a medium-sized educational district in western Saudi Arabia. To comply with gender-segregated schooling practices, a male researcher collected data in boys’ schools and a female researcher in girls’ schools. Working in parallel, they administered the questionnaires in scheduled class periods, each requiring approximately 20 min. On average, one to two class groups were surveyed per school, and the 15-day window provided sufficient buffer time for replacements and make-up sessions, ensuring the final sample of 300 valid responses (150 males, 150 females).

### 3.5 Data analysis

Data were analyzed using several statistical techniques aligned with the TPB framework as a psychological model. Because all constructs were measured using student self-report questionnaires administered at a single time point, the possibility of common method bias was systematically assessed. Procedural remedies included the use of previously validated and piloted scales with clearly separated subscales for each construct, assurance of participant anonymity, and gender-segregated administration by trained researchers. In addition, Harman’s single-factor test was conducted by entering all questionnaire items into an unrotated principal component analysis. The results of Harman’s single-factor test are presented in Table 1. The first unrotated factor accounted for 41.3% of the total variance (eigenvalue = 14.44), which is below the conventional heuristic 50% threshold commonly used to indicate substantial common method bias. These results suggest that no single factor dominated the covariance structure among the measures, and that common method variance is unlikely to represent a substantial threat to the validity of the findings. Nevertheless, future research should employ multi-method or longitudinal designs to more rigorously evaluate this issue.

**TABLE 1:** Harman’s single-factor test results.

Factor	Eigenvalue	Variance explained %	Cumulative %
1	14.44	41.3	41.3
2	1.87	5.3	46.6
3	0.64	1.8	48.4

The first unrotated factor explained 41.3% of the total variance, which is below the 50% threshold commonly used to indicate substantial common method bias.

Pearson’s correlation examined associations among study variables. Independent samples *t*-tests compared male and female students.

Multiple regression analysis tested the predictive roles of teachers’ innovative knowledge and classroom climate on students’ innovative attitudes. Mediation analysis (Baron and Kenny, 1986) assessed the indirect effect of classroom climate, with further evaluation via the Sobel test and calculation of the Proportion Mediated (PM) as an effect size. Mediation was examined following the Baron and Kenny (1986) procedure supplemented by the Sobel test. Although contemporary approaches often recommend bootstrapped confidence intervals for estimating indirect effects, the present analysis followed this classical procedure, which remains widely used in educational research. This approach was adopted to maintain comparability with prior educational studies that have applied the Baron and Kenny framework in examining mediation relationships. Future studies may employ bootstrapping to obtain more robust estimates of mediation effects. Accordingly, the indirect pathways identified in this study should be interpreted as preliminary associational evidence rather than definitive causal mediation.

Finally, moderation analysis using hierarchical regression determined whether gender conditioned the strength of these pathways. Together, these analyses illuminated the psychological mechanisms (norms, perceived control, and attitudes) underlying students’ innovative dispositions in mathematics classrooms.

## 4 Results

Analyses were based on data from 300 first-grade secondary students (150 males, 150 females) enrolled in public schools within a medium-sized educational district in western Saudi Arabia. All statistical procedures were conducted using standard quantitative analysis techniques, and results are presented in line with the study’s four hypotheses (H1–H4), beginning with descriptive statistics and followed by tests of association, prediction, mediation, and moderation.

### 4.1 Descriptive statistics

As shown in Table 2, female students reported higher mean scores than males across all psychological–educational constructs: perceptions of teachers’ innovative pedagogical knowledge, innovative classroom climate, and their own innovative attitudes. These differences suggest that female students may be more sensitive to innovation-oriented socio-emotional cues, providing justification for testing gendered pathways in subsequent analyses.

**TABLE 2:** Means and standard deviations of study variables by gender (*N* = 300).

Variable	Gender	*n*	M	SD
Teachers’ innovative knowledge	Male	150	3.89	0.72
	Female	150	4.12	0.67
Innovative classroom climate	Male	150	3.70	0.78
	Female	150	3.90	0.73
Students’ innovative attitudes	Male	150	3.80	0.70
	Female	150	4.10	0.68

Scale anchors: 1 = Strongly Disagree to 5 = Strongly Agree.

### 4.2 Testing H1: association between teachers’ innovative knowledge and students’ innovative attitudes

Pearson’s correlation analyses examined the association between students’ perceptions of their teachers’ innovative pedagogical knowledge and their own innovative attitudes. Results, summarized in Table 3, indicated significant positive correlations for both male and female students. Among boys, the association was moderate (*r* = 0.42, *p* < 0.01), whereas among girls the correlation was stronger (*r* = 0.58, *p* < 0.01).

**TABLE 3:** Correlation between perceived innovative knowledge and innovative attitudes by gender.

Gender	*n*	*r*	*p*
Male	150	0.42	< 0.01
Female	150	0.58	< 0.01

These findings provide strong support for H1. They suggest that when teachers apply innovative pedagogical practices, students are more likely to develop positive attitudes toward innovation in mathematics. Importantly, the effect was more pronounced among female students, highlighting gender differences in psychological responsiveness to teachers’ innovative knowledge.

### 4.3 Testing H2: mediation of classroom climate

Correlation and regression analyses assessed the associations between teachers’ innovative knowledge and students’ innovative attitudes. For females, all pathways were significant: innovative knowledge was associated with both classroom climate (β = 0.40, *p* < 0.01) and students’ innovative attitudes (β = 0.45, *p* < 0.01); in turn, classroom climate was associated with students’ attitudes (β = 0.37, *p* < 0.01). When classroom climate was added to the model, the direct effect of innovative knowledge decreased (β = 0.35), suggesting a potential indirect pathway. The Sobel test indicated a pattern consistent with an indirect effect; however, this should be interpreted cautiously (*Z* = 3.12, *p* < 0.01), with approximately 25% of the total association associated with this potential indirect pathway through classroom climate.

For males, innovative knowledge was associated with classroom climate (β = 0.25, *p* < 0.01) and attitudes (β = 0.29, *p* < 0.01), but classroom climate did not significantly predict attitudes (β≈ 0, *p* > 0.05). Thus, no evidence of an indirect pathway was found for the male group. These results are presented in Table 4.

**TABLE 4:** Correlations and regression coefficients for mediation analysis by gender.

Gender	Path	*r*	*p* (r)	*B*	*F*	df	p (β)
Female	X → Y (c)	0.58	<0.01	0.450	35.87	1,148	<0.01
	X → M (a)	0.50	<0.01	0.400	49.33	1,148	<0.01
	M → Y (b)	0.39	<0.01	0.371	12.65	1,148	<0.01
	X → Y (c′)	–	–	0.350	24.91	2,147	<0.01
Male	X → Y (c)	0.42	<0.01	0.294	12.34	1,148	<0.01
	X → M (a)	0.30	<0.01	0.250	14.67	1,148	<0.01
	M → Y (b)	0.18	0.06	0.001	1.82	1,148	0.18
	X → Y (c′)	–	–	0.294	6.21	2,147	<0.01

X = Teachers’ innovative pedagogical knowledge; M = Classroom climate; Y = Students’ innovative attitudes. Paths follow Baron and Kenny’s (1986) mediation framework: (c) total effect of X on Y; (a) effect of X on M; (b) effect of M on Y; (c′) direct effect of X on Y after controlling for M.

**FIGURE 2:**
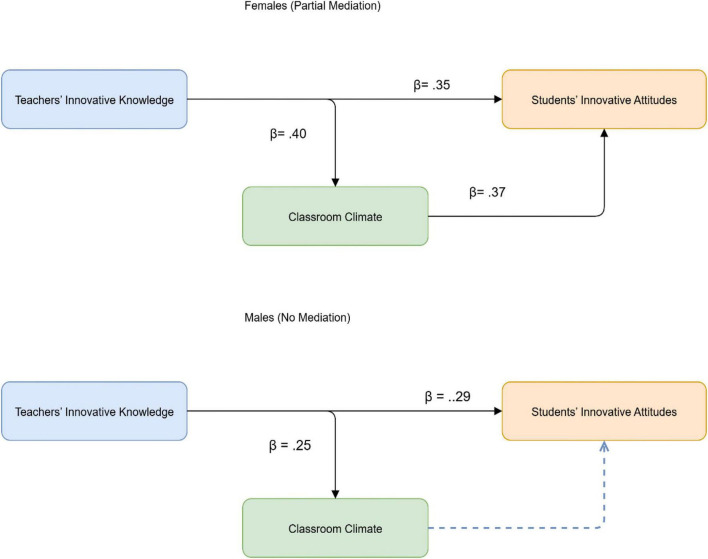
Mediation models for female and male students.

Overall, H2 was supported in a qualified sense for females only, with the pattern of results being consistent with a potential indirect pathway through classroom climate suggesting a pattern of association between teachers’ innovative practices and female students’ innovative attitudes. For males, however, teachers’ innovative knowledge remained the primary factor associated with students’ innovative attitudes, with no evidence of an indirect pathway observed. These mediation pathways are illustrated in Figure 2.

For female students (left), classroom climate was associated with a potential indirect pathway between teachers’ innovative pedagogical knowledge and students’ innovative attitudes, with approximately 25% of the total effect associated with this potential the indirect pathway. For male students (right), the mediating effect of classroom climate was non-significant, and teachers’ innovative knowledge remained the sole predictor of students’ innovative attitudes.

### 4.4 Testing H3: gender differences in perceptions of innovative knowledge

An independent samples *t*-test compared male and female students’ perceptions of their teachers’ innovative pedagogical knowledge. Results showed that females reported significantly higher scores (M = 4.12, SD = 0.67) than males (M = 3.89, SD = 0.72), *t*(298) = 2.87, *p* = 0.004, Cohen’s d = 0.33, 95% CI [0.07, 0.39].

This finding supports H3, suggesting that female students perceived stronger normative signals of innovation from their teachers, reflecting gendered differences in perception and responsiveness. Details are provided in Table 5.

**TABLE 5:** Independent samples *t*-test comparing male and female students’ perceptions of teachers’ innovative knowledge.

Gender	*N*	*M*	SD	*t*	Df	*p*	Cohen’s *d*	95% CI
Male	150	3.89	0.72					
Female	150	4.12	0.67	2.87	298	0.004	0.33	[0.07, 0.39]

**FIGURE 3:**
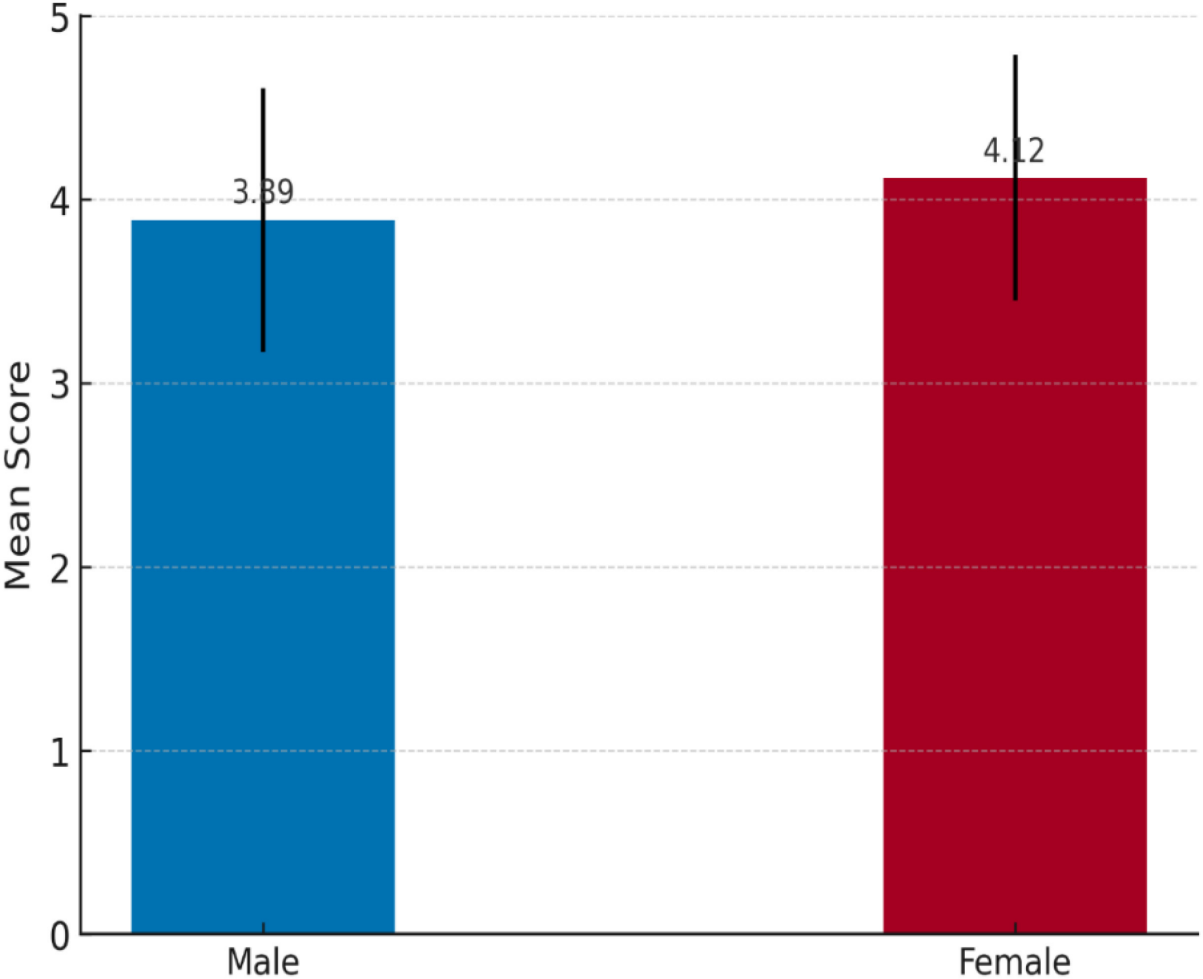
Gender differences in perceptions of teachers’ innovative pedagogical knowledge. Bar chart with error bars ( ± 1 SE) shows significantly higher female scores (*p* < 0.01, *d* = 0.33).

Figure 3 illustrates this gender difference, with females consistently reporting more favorable perceptions of their teachers’ innovative knowledge than males.

### 4.5 Testing H4: predictive contribution of innovative knowledge and classroom climate

Multiple regression analyses were conducted to determine how teachers’ innovative pedagogical knowledge and classroom climate predicted students’ innovative attitudes, with results reported separately for male and female students.

For male students, innovative knowledge was the only significant predictor (β = 0.29, *p* < 0.01). It explained 7.7% of the variance in innovative attitudes. Classroom climate, by contrast, did not contribute significantly. When both predictors were entered together, the model accounted for just 7.8% of the variance, confirming that innovative knowledge was the sole driver of male students’ innovative attitudes. These results are summarized in Table 6.

**TABLE 6:** Regression predicting innovative attitudes for male students.

Predictor	*R* ^2^	β	95% CI for β	*F*	df	*p*
Innovative knowledge	0.077	0.294	[0.130, 0.458]	12.34	1,148	< 0.01
Innovative climate	0.012	0.001	[–0.001, 0.003]	1.82	1,148	0.18
Combined model	0.078	–	–	6.21	2,147	< 0.01

*R*^2^ = proportion of variance explained in students’ innovative attitudes.

The pattern was different for female students. Here, both predictors were significant. Innovative knowledge predicted innovative attitudes (β = 0.45, *p* < 0.01), explaining 19.5% of the variance. Classroom climate also played a meaningful role (β = 0.37, *p* < 0.01), adding another 7.9%. In the combined model, both variables remained significant, with innovative knowledge (β = 0.35, *p* < 0.01) and classroom climate (β = 0.20, *p* < 0.05) jointly accounting for 25.3% of the variance. These effect sizes indicate that innovative teaching practices and supportive classroom environments account for a meaningful share of the variation in students’ innovative attitudes toward mathematics learning. These findings are reported in Table 7.

**TABLE 7:** Regression predicting innovative attitudes for female students.

Predictor	*R* ^2^	β	95% CI for β	*F*	df	*p*
Innovative knowledge	0.195	0.450	[0.303, 0.597]	35.87	1,148	< 0.01
Innovative climate	0.079	0.371	[0.167, 0.575]	12.65	1,148	< 0.01
Combined model	0.253	–	–	24.91	2,147	< 0.01
–Knowledge	–	0.350	[0.203, 0.497]	–	–	< 0.01
–Climate	–	0.200	[0.036, 0.364]	–	–	< 0.05

*R*^2^ = proportion of variance explained in students’ innovative attitudes. CI, confidence interval. Both predictors (teachers’ innovative knowledge and classroom climate) remained significant in the combined model, jointly explaining 25.3% of the variance.

**FIGURE 4:**
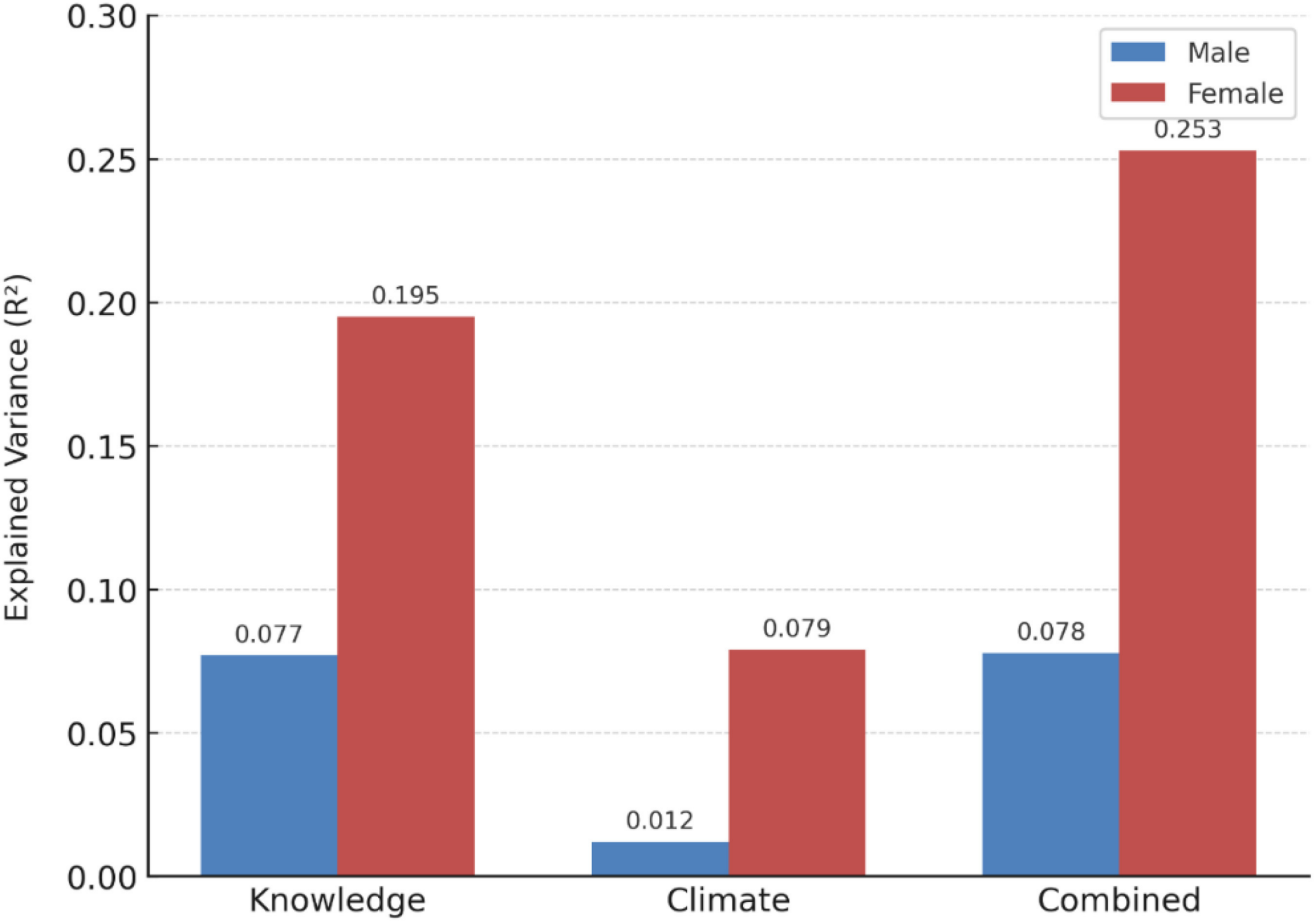
Explained variance (*R*^2^) in students’ innovative attitudes by gender and model.

These findings confirm H4, indicating that teachers’ innovative pedagogical knowledge and classroom climate jointly predict students’ innovative attitudes, with much stronger effects observed among female students. While male students’ attitudes were driven solely by teacher innovation, female students’ attitudes reflected a synergistic influence of both teacher practices and supportive classroom environments. This pattern is illustrated in Figure 4.

Bar chart shows that among males, teacher innovative knowledge explained modest variance (*R*^2^ = 0.077), with negligible contribution from classroom climate and minimal gain in the combined model (*R*^2^ = 0.078). Among females, both predictors contributed meaningfully (*R*^2^ = 0.195 for knowledge; *R*^2^ = 0.079 for climate), yielding the highest explained variance in the combined model (*R*^2^ = 0.253).

### 4.6 Moderation by gender

Hierarchical regression analyses were conducted to test whether gender moderated the influence of teachers’ innovative pedagogical knowledge and classroom climate on students’ innovative attitudes. The results are reported in Table 8.

**TABLE 8:** Hierarchical regression testing gender interactions.

Step	*R* ^2^	Δ *R*^2^	Predictor	β	95% CI for β	*F*	df	*p*
1	0.142	–	Gender	0.100	[0.020, 0.180]	24.58	2,297	< 0.05
			Innovative knowledge	0.370	[0.250, 0.490]			< 0.01
2	0.163	0.021	Gender × knowledge	0.150	[0.060, 0.240]	10.45	3,296	< 0.01
1	0.050	–	Gender	0.080	[0.010, 0.150]	7.82	2,297	< 0.05
			Innovative climate	0.200	[0.080, 0.320]			< 1.01
2	0.065	0.015	Gender × climate	0.120	[0.038, 0.202]	8.00	3,296	< 0.05

Interaction terms (Gender × Knowledge, Gender × Climate) test moderation effects. Δ*R*^2^ = change in explained variance after adding the interaction term. Significant Δ*R*^2^ values indicate improved model fit.

The interaction between gender and innovative knowledge reached significance (β = 0.15, *p* < 0.01). This means that the positive effect of teacher knowledge was stronger for female students than for males. A similar pattern appeared for classroom climate. The interaction was significant (β = 0.12, *p* < 0.05), showing that girls benefited more from supportive environments than boys.

**FIGURE 5:**
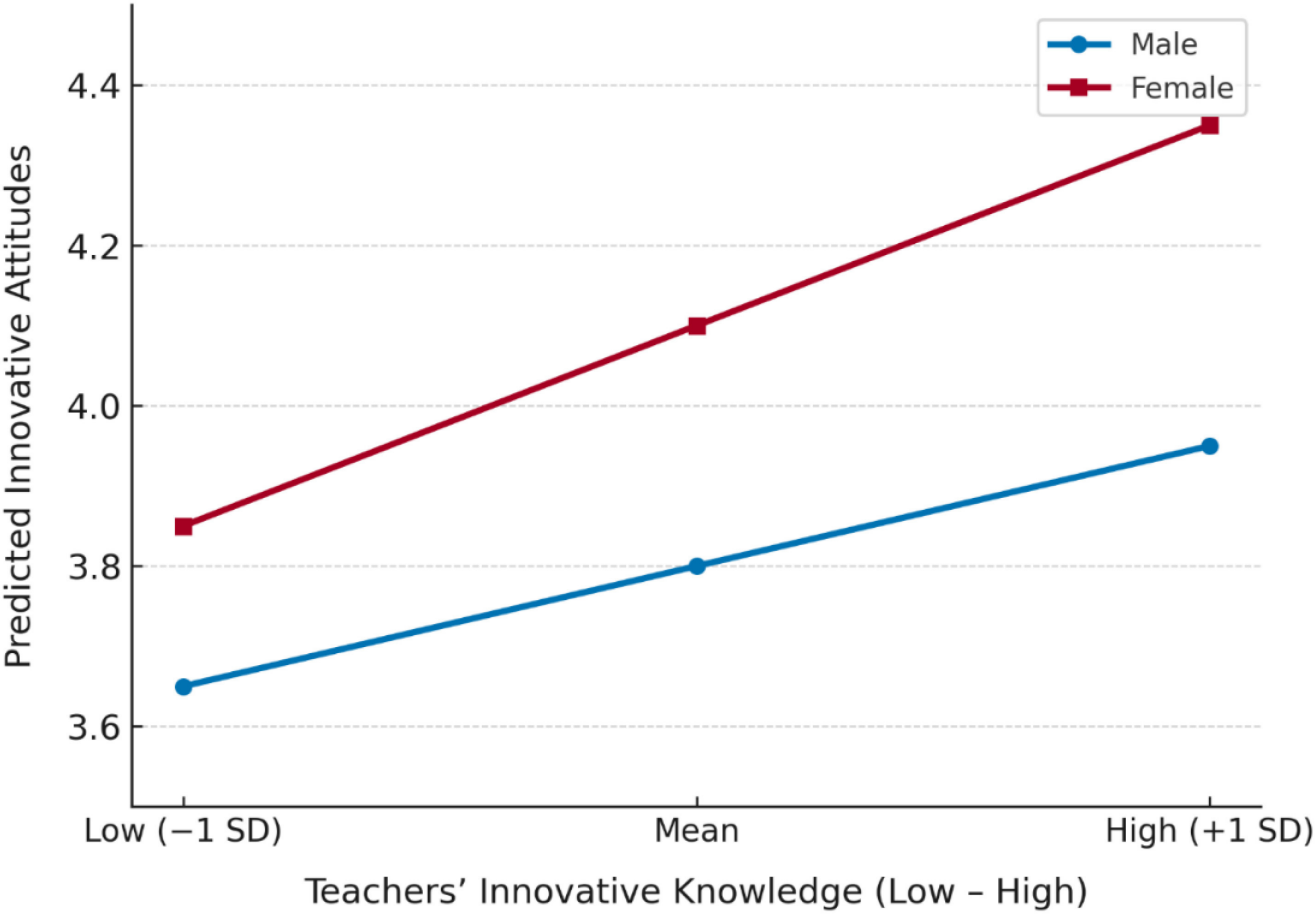
Interaction between gender and innovative knowledge in predicting students’ innovative attitudes.

**FIGURE 6:**
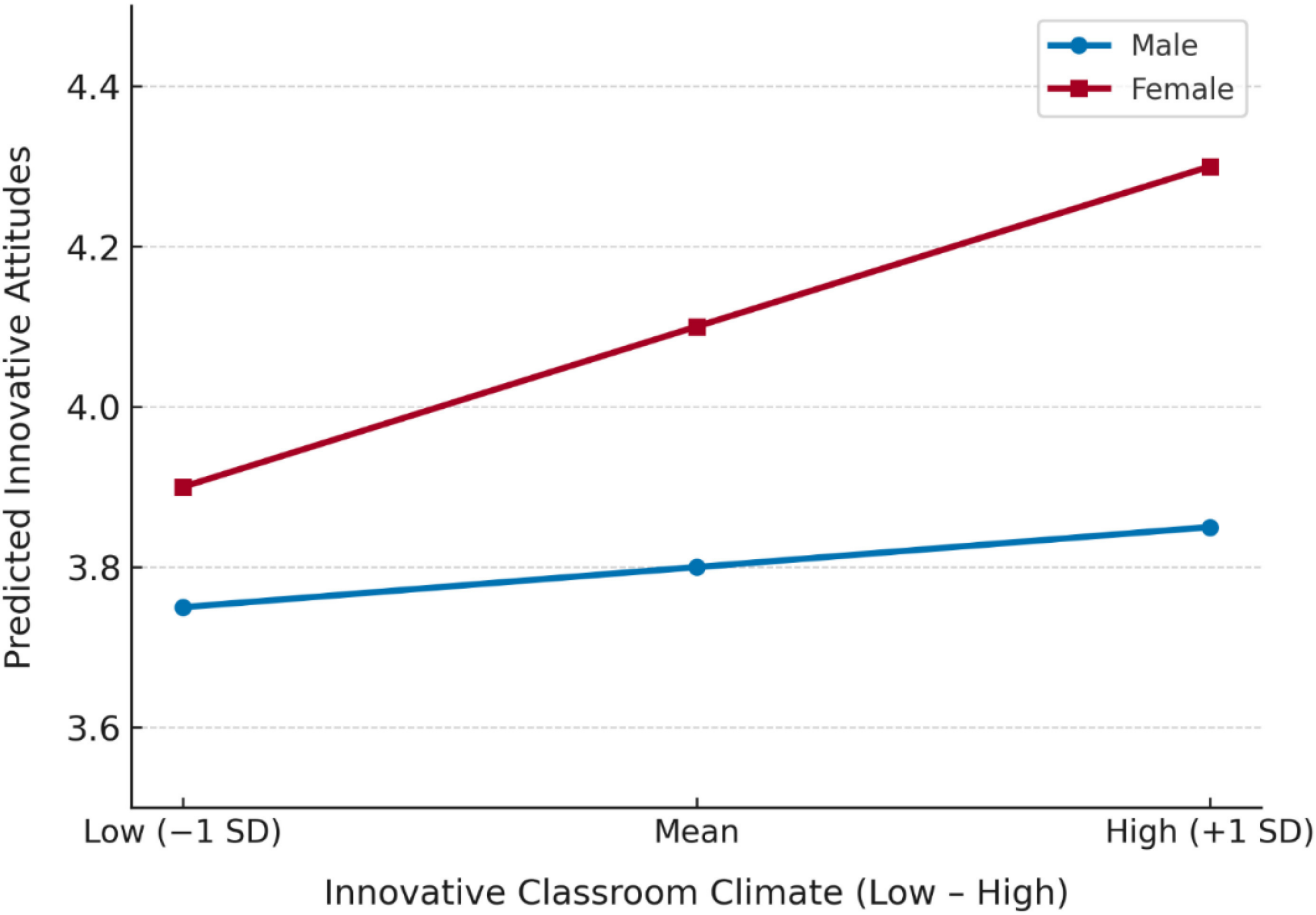
Interaction between gender and innovative classroom climate in predicting students’ innovative attitudes.

Figures 5, 6 illustrate these effects, with steeper slopes for females highlighting how gender shaped the strength of both predictors.

The slope for females is steeper, indicating that higher perceptions of teachers’ innovative knowledge are associated with a stronger increase in innovative attitudes among female students compared to males.

Female students’ innovative attitudes increase more markedly in supportive classroom climates, while the effect of climate is negligible for male students. Together, these findings provide strong support for H3, showing that gender significantly moderated both relationships, with consistently stronger effects for females.

In summary, the analyses supported all four hypotheses. Teachers’ innovative knowledge consistently predicted students’ innovative attitudes, while classroom climate was associated with a potential indirect pathway among female students only. Gender emerged as a significant moderator, with females perceiving higher innovative knowledge and deriving stronger benefits from both teacher knowledge and supportive climates. The combined predictive model explained substantially more variance for females than for males, setting the stage for the discussion of gendered dynamics in mathematics classrooms.

## 5 Discussion and conclusion

### 5.1 Overview of key findings

The findings of this study shed light on how teachers’ innovative pedagogical knowledge and classroom climate are associated with students’ innovative attitudes in mathematics, while also revealing clear gender-based dynamics. Across the full sample, teachers’ innovative knowledge consistently stood out as a significant predictor, confirming its central role in fostering creativity-oriented dispositions.

However, the strength of this relationship varied by gender. Female students showed greater responsiveness to innovative practices than their male peers. For them, classroom climate was associated with a potential indirect pathway: supportive, discussion-oriented, and exploratory environments appeared to support the association between teacher innovation and students’ innovative attitudes. By contrast, no such mediation was observed among male students.

Gender further moderated both pathways. The predictive power of teachers’ knowledge and classroom climate was amplified for females, whereas male students’ attitudes appeared to depend mainly on teacher practices alone. Taken together, these results suggest that innovative teaching is a key factor associated with students’ dispositions, but the mechanisms through which it operates are not uniform. They highlight the particular importance of supportive classroom environments for female learners.

### 5.2 Interpreting findings through the theory of planned behavior

This study drew on the Theory of Planned Behavior (TPB) (Ajzen, 1991), which highlights how attitudes, social norms, and perceived control interact to shape behavior. In this framework, teachers’ innovative pedagogical knowledge functioned as a social norm, sending a signal to students that creativity and innovation are valued in mathematics learning. Classroom climate captured perceived control, showing the extent to which students felt supported in exploring ideas and trying non-traditional approaches. Students’ innovative attitudes, in turn, reflected the attitudinal outcome that TPB seeks to explain.

The results aligned with this framework but also revealed important differences. Teachers’ innovative knowledge predicted students’ innovative attitudes for male and female students, confirming the strong normative role teachers play. At the same time, classroom climate was associated with an pattern consistent with a potential indirect pathway only for female students. For them, supportive and open environments may help explain the association between teacher innovation and students’ attitudes. For male students, this link was absent, suggesting that their responses depended more directly on teacher practices.

This pattern echoes the view that TPB pathways are shaped by context (Eriksson, 2020). It also mirrors recent findings in mathematics education. Innovative classroom climates have been shown to strengthen problem-solving, especially among female students (Lu et al., 2025). Other studies (Zhang and He, 2024) demonstrated that socio-emotional resources play a greater role for female learners when engaging in creative mathematical tasks. Further evidence (Krejcie and Morgan, 1970) reinforces this view, showing that mathematical creativity in modeling flourishes when students are given supportive environments that enable not only novel solutions but also the reformulation of problems into meaningful models, underscoring the role of classroom climate as a catalyst for innovation.

Taken together, these findings confirm the usefulness of TPB while also cautioning against a one-size-fits-all interpretation. The mechanisms that drive students’ innovative attitudes depend not only on teachers’ practices but also on gendered responses to the classroom environment. This underscores the importance of examining TPB through cultural and gender-sensitive lenses. In this interpretation, teachers’ innovative pedagogical knowledge may function as a normative influence, while classroom climate represents a contextual form of perceived behavioral control that supports the development of innovative attitudes. These findings contribute to extending the application of the Theory of Planned Behavior in mathematics education by demonstrating how instructional practices and classroom environments jointly shape students’ innovative attitudes within gender-segregated educational contexts.

### 5.3 Gendered dynamics in innovative learning

One of the most notable findings of this study is that female students appeared more responsive to the quality of classroom climate than their male peers. For boys, innovative attitudes were influenced mainly by how they viewed their teachers’ innovative knowledge. Girls, however, showed greater benefit when teaching was paired with environments that felt supportive, collaborative, and open to exploration.

This result mirrors international evidence. Studies have shown that female learners often attach more importance to relational and environmental supports in learning contexts (Zhang and He, 2024; Lu et al., 2025). Other findings (Lu et al., 2025) likewise demonstrated that innovative classroom climates boost problem-solving capacity more strongly among girls. Additional evidence (Zhang and He, 2024) indicated that socio-emotional resources play a disproportionate role in sustaining female engagement in creative tasks.

In Saudi Arabia, the system of gender-segregated schooling may further reinforce these dynamics. Female students’ attitudes appear shaped not only by their teachers’ practices but also by the wider classroom atmosphere, where collaboration among peers and teacher–student relationships carry extra weight. Male students, by contrast, seem to rely more directly on instructional cues, aligning with research that suggests boys are less dependent on relational or socio-emotional supports in their academic engagement.

These findings converge with global reports (Torrance, 1964), which emphasize gendered differences in how supportive climates nurture creativity and innovation. Together, they highlight the importance of designing pedagogical and policy strategies that explicitly address gender in order to create equitable opportunities for innovation. These differences should not be interpreted as inherent or fixed gender traits, but rather as context-dependent patterns shaped by classroom environments and sociocultural expectations.

### 5.4 Predictive contributions and theoretical refinement

The regression analyses further clarified how teachers’ innovative knowledge and classroom climate interact in shaping students’ innovative attitudes. Among female students, the two predictors jointly explained more than one-quarter of the variance, highlighting that their innovative dispositions emerge from a synergy between teacher practices and a supportive environment. For male students, however, innovative knowledge alone was sufficient to predict attitudes, while classroom climate added no explanatory power. This contrast suggests that teacher-driven innovation is an important factor associated with students’ dispositions, but the extent to which classroom environments enhance this effect is gender-contingent.

From a theoretical perspective, these findings refine the application of the Theory of Planned Behavior (TPB) by showing that perceived behavioral control, in this case classroom climate, does not operate uniformly across groups. Gender acts as a moderator that may influence how environmental resources are associated with students’ innovative attitudes. This is consistent with the observation that teachers’ creative pedagogical knowledge alone does not guarantee its enactment; rather, its effectiveness depends on whether classroom conditions support or constrain innovative practices (Xie and Liu, 2023).

Rather than challenging the validity of TPB, the results offer a contextualized extension: behavioral models remain robust, but their predictive pathways must be interpreted through cultural and gender-specific educational realities. Recent evidence in mathematics education reinforces this point, showing that contextual supports amplify innovation outcomes more strongly among female learners than their male peers (Lu et al., 2025). Similarly, other findings (Zhang and He, 2024) demonstrated that gendered differences in responsiveness to learning environments play a pivotal role in shaping students’ academic dispositions, with females benefiting more from supportive and relational climates. Thus, the contribution of this study lies not only in confirming the power of teacher innovation but also in demonstrating that its association with students’ innovative attitudes may vary according to the broader classroom environment and shaped by gendered dynamics. Future research may further examine these relationships using structural equation modeling (SEM) to test the full set of pathways within a latent-variable framework.

### 5.5 Practical and policy implications

The findings of this study point to several implications for both practice and policy. First, the consistent impact of teachers’ innovative knowledge highlights the need for focused professional development. Programs should equip teachers with the skills to design creative lessons, reformulate mathematical problems in new ways, and make purposeful use of digital tools. Prior research supports this direction, showing that such practices strengthen students’ dispositions toward innovation across a range of contexts (Alshahrani and Ally, 2016; Rodríguez et al., 2020; Nkundabakura et al., 2023).

Second, the gender differences observed in this study underline the importance of gender-responsive classrooms. Because female students were especially sensitive to the quality of classroom climate, schools should prioritize environments that are collaborative, inclusive, and supportive. Normalizing experimentation and group problem-solving can make innovation feel accessible and natural, particularly for girls.

Third, classroom climate itself needs to be monitored more systematically. Tools such as student feedback surveys can help teachers and school leaders assess whether learning environments are effectively fostering innovation and whether certain groups of learners, especially females, are receiving the support they need.

Finally, education policy aimed at strengthening STEM education should recognize that teacher innovation and classroom climate work best when treated as complementary levers. Effective strategies will be those that not only invest in teachers’ pedagogical creativity but also account for the different ways male and female students respond to their classroom environments.

### 5.6 Limitations and final remarks

Several limitations should be acknowledged. First, the cross-sectional design restricts the ability to draw causal inferences; longitudinal or experimental studies would provide stronger evidence for developmental trajectories and causal mechanisms.

Second, the reliance on student self-reports raises concerns about potential response biases. Future research should incorporate multiple data sources, such as teacher assessments and systematic classroom observations, to enhance validity. Because the study relied exclusively on student self-reports collected at a single time point, common method variance may have influenced the strength of some observed relationships. Harman’s single-factor test indicated that no single factor dominated the variance, and procedural remedies (as detailed in section 3.5) were implemented to mitigate this concern. Nevertheless, shared method bias cannot be entirely ruled out and may have influenced some of the observed associations. Future research may benefit from using multiple data sources or longitudinal designs to further reduce this concern. Accordingly, the indirect pathways identified in this study should be interpreted with caution as preliminary associational evidence rather than definitive causal mediation findings. Future research may also benefit from applying confirmatory factor analysis (CFA) to further examine the measurement structure of the constructs and strengthen evidence of construct validity.

Third, the study was conducted in a single Saudi city, which limits the generalizability of findings. Replication across diverse regions and educational systems would help establish broader applicability. Finally, future studies should test additional moderating variables, including socioeconomic status, prior academic achievement, and teacher gender, to extend insights offered by comparative research (Alnahdi and Schwab, 2023) and to build a more nuanced understanding of the contextual factors shaping innovative attitudes in mathematics education.

In conclusion, this study contributes to the growing body of research by showing how teachers’ innovative pedagogical knowledge and the classroom climate are jointly associated with students’ innovative attitudes, with gender playing a key moderating role. Viewed through the lens of the Theory of Planned Behavior, the findings indicate that innovation in mathematics education is associated with both instructional practices and socio-emotional classroom conditions. The results highlight the importance of gender-responsive pedagogy and supportive learning environments in fostering students’ innovative dispositions.

## Data Availability

The raw data supporting the conclusions of this article will be made available by the authors, without undue reservation.
